# Hemorrhagic Myocardial Infarction Implicating Left Ventricular Thrombus

**DOI:** 10.1016/j.jaccas.2026.107612

**Published:** 2026-03-25

**Authors:** Keyur P. Vora, Rohan Dharmakumar

**Affiliations:** a Cardiovascular Imaging Research Center, Medical Imaging Research Institute, Indiana University School of Medicine, Indianapolis, Indiana, USA; b Division of Cardiovascular Medicine, Department of Medicine, Indiana University School of Medicine, Indianapolis, Indiana, USA

**Keywords:** hemorrhage, myocardial infarction, myocardial revascularization, thrombus

## Abstract

**Background:**

Left ventricular thrombus formation following ST-segment elevation myocardial infarction is typically attributed to blood stasis in an akinetic apex. However, thrombus can form even in the presence of anticoagulation, suggesting alternative mechanisms may be at play.

**Case Summary:**

A 44-year-old man with an anterior ST-segment elevation myocardial infarction underwent primary percutaneous coronary intervention with successful restoration of TIMI flow grade 3. Despite receiving 19,000 U of heparin, a mobile apical thrombus formed. Cardiac magnetic resonance revealed a large intramyocardial hemorrhage. Feature-tracking strain analysis quantified a “mechanical tug of war” at the infarct border. The magnitude of the shear was spatially concordant with a suspected endocardial disruption, creating structural nidus for thrombosis.

**Discussion:**

This case demonstrates that severe reperfusion intramyocardial hemorrhage can induce a mechanical breach of the endocardium. Recognition of hemorrhagic dissection via cardiac magnetic resonance shifts the management paradigm from treating generic stasis to managing a specific structural failure.

## Graphical Abstract

**Figure:**
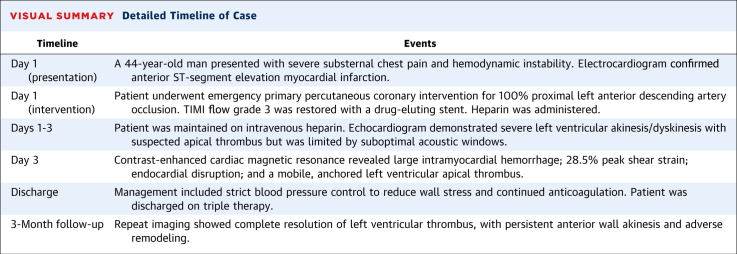

## History of Presentation

A 44-year-old man presented to the emergency department with sudden onset of severe substernal chest pain and palpitations. He arrived within 30 minutes of symptom onset. On physical examination, he exhibited signs of hemodynamic instability, including tachycardia (heart rate 110 beats/min) and hypotension (blood pressure 90/60 mm Hg), with cool extremities consistent with incipient cardiogenic shock.

**Take-Home Messages**

• IMH is associated with high peri-infarct dyssynchrony, which can compromise myocardial integrity.
• Structural endocardial disruption can serve as an anchor for thrombus.
• CMR feature tracking offers mechanistic insights into post-MI complications often invisible on echocardiography.

## Past Medical History

His medical history was notable for a 27-pack-year smoking history, class I obesity (body mass index 38.5 kg/m^2^), and newly diagnosed diabetes mellitus and hypertension. He had no prior history of coagulopathy, thromboembolic events, or atrial fibrillation.

## Differential Diagnosis

Given the acute presentation of chest pain with hemodynamic instability, the differential diagnosis included acute coronary syndrome, acute aortic dissection, and massive pulmonary embolism. A 12-lead electrocardiogram showing distinct ST-segment elevation in the anterior precordial leads confirmed the diagnosis of anterior wall ST-segment elevation myocardial infarction (STEMI).

## Investigations

**Figure 1:**
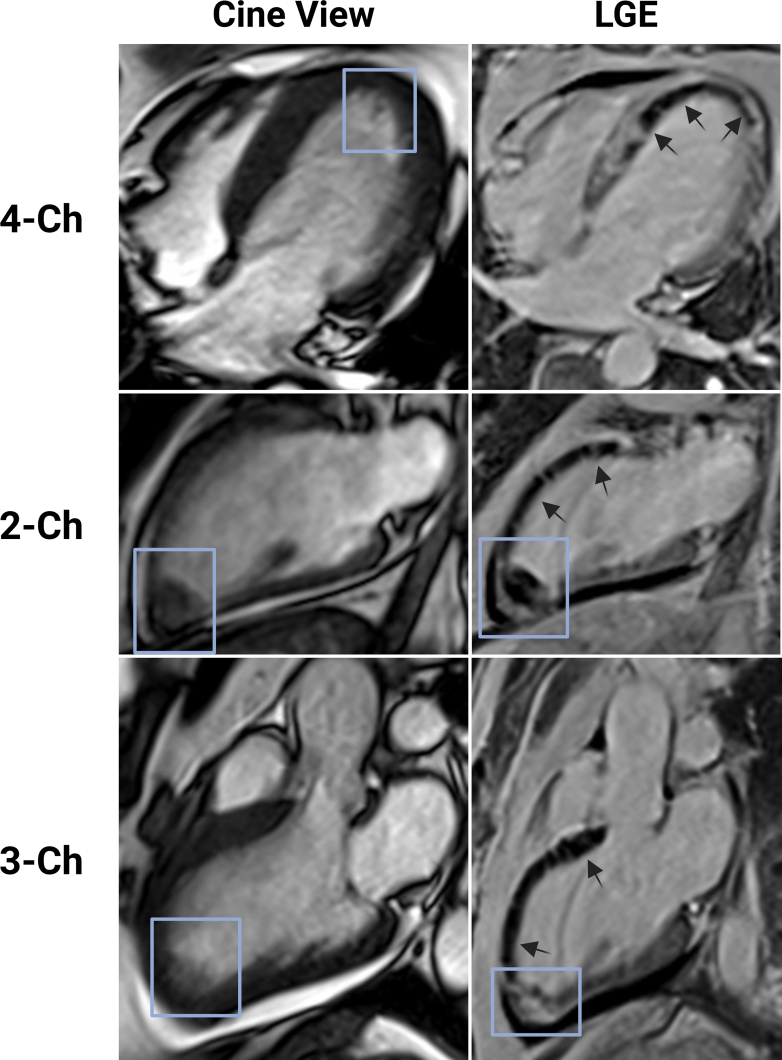
Tissue Characterization and Left Ventricular Thrombus Matched end-diastolic cine (left column) and late gadolinium enhancement (LGE) (right column) cardiovascular magnetic resonance images are shown in 4-chamber, 2-chamber, and 3-chamber long-axis views. Cine imaging reveals a distinct, mobile, low-signal-intensity mass consistent with a thrombus attached to the dyskinetic left ventricular apex (blue boxes). The corresponding LGE images demonstrate extensive transmural infarction (bright signal) of the anterior and septal walls. Black arrows indicate a substantial subendocardial zone of hypoenhancement (dark core) representing persistent microvascular obstruction, which extends to the endocardial border precisely at the site of thrombus attachment and is a sign of significant microvascular damage within the infarct zone. Ch = chamber.

**Figure 2:**
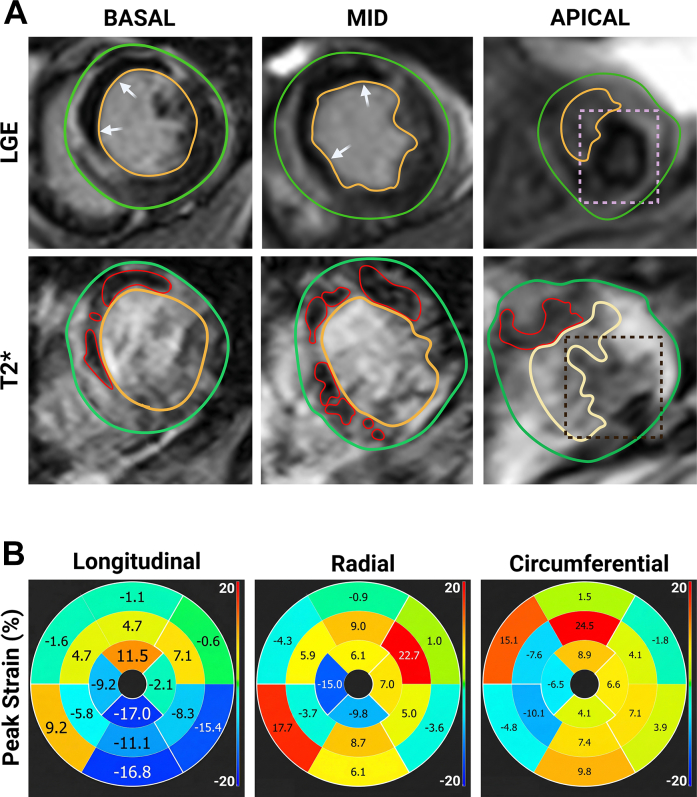
Tissue Characterization and Biomechanical Analysis (A) Short-axis images at basal, midventricular, and apical levels characterizing the infarct substrate are shown. Late gadolinium enhancement (LGE) (top row) delineates the transmural infarct containing a central hypointense core of persistent microvascular obstruction (white arrows). T2∗ (bottom row) maps confirm that the persistent microvascular obstruction is representative of intramyocardial hemorrhage, characterized by a distinct signal void (red outlines). Note that severe intramyocardial hemorrhage can obscure the precise identification of the endocardial border, though the signal void in this case is spatially concordant with the region of injury and thrombus anchoring. (B) Feature-tracking strain maps shows the “mechanical tug of war” between healthy and infarcted hemorrhagic myocardium. The longitudinal strain map (left) helps to visualize this critical mechanical mismatch: the remote healthy myocardium (blue segments) exhibits hyperdynamic shortening (negative strain −17.0%), pulling vigorously against the hemorrhagic infarct core (orange segments), which undergoes paradoxical systolic stretching (positive strain +11.5%). These directly opposing motion vectors create a peak shear strain of 28.5% at the border zone. This extreme shearing force contributes to the mechanical disruption of the fragile endocardium, creating the structural breach potentially serving as the nidus for thrombosis.

Emergency coronary angiography revealed a proximal 100% occlusion of the left anterior descending artery with a high angiographic thrombus burden. To assess myocardial viability and the extent of reperfusion injury, a contrast-enhanced cardiac magnetic resonance (CMR) scan was performed on hospital day 3. The scan revealed severe LV dysfunction (LV ejection fraction 27.6%) with akinesis of the entire anterior wall and septum. Late gadolinium enhancement imaging demonstrated transmural infarction affecting nearly 68% of the total LV mass. Notably, a large subendocardial zone of hypoenhancement was visualized within the infarct core, consistent with persistent microvascular obstruction (Figure 1). T2∗ mapping corroborated this finding, revealing a distinct hypointense core confirming massive intramyocardial hemorrhage (IMH) (Figure 2A).

### Feature tracking analysis

CMR feature tracking quantified the mechanical severity of the injury. The strain maps revealed a distinct mechanical mismatch (Figure 2B): the remote inferior myocardium exhibited hyperdynamic shortening (longitudinal strain −17.0%), whereas the hemorrhagic anterior wall exhibited profound systolic dyskinesis (longitudinal strain +11.5%). This differential motion generated a peri-infarct shear stress magnitude of 28.5%, confirming abnormal stretching of the necrotic core during systole.

### Thrombus characterization

CMR identified an irregular, elongated, and mobile mass at the LV apex measuring 18.6 mm × 14.7 mm × 10.0 mm (Video 1, Video 2, Video 3). Spatially, the thrombus was anchored directly to the area of maximal wall thinning and endocardial disruption caused by the IMH (Figure 1).

## Management

On presentation, the patient received a loading dose of dual antiplatelet therapy (aspirin 325 mg, clopidogrel 300 mg), a high-intensity statin, and an initial heparin bolus of 5,000 U. During primary percutaneous coronary intervention (PCI), a single drug-eluting stent (3.5 × 28 mm) was deployed. Due to the heavy thrombotic burden, the patient required a cumulative intraprocedural heparin dose of 14,000 U to maintain therapeutic activated clotting times. Although TIMI flow grade 3 was restored, the procedure was complicated by a brief episode of ventricular tachycardia and slow flow requiring continuous nicorandil infusion.

Post-procedurally, the patient received intravenous heparin 5,000 U every 6 hours for 72 hours, followed by a transition to subcutaneous enoxaparin (1 mg/kg every 12 hours). As a post-reperfusion echocardiogram at 48 hours did not definitively confirm a thrombus (Video 4, Video 5, Video 6, Video 7) with suboptimal acoustic windows, the patient was maintained on this specific regimen. However, the unique morphology and persistence of the thrombus observed on CMR suggested a structural component anchored by the hemorrhagic dissection.

Following the CMR diagnosis of LV thrombus and hemorrhagic dissection, the patient was managed with strict blood pressure control to limit wall stress (Laplace's law). Anticoagulation was continued to manage the mobile thrombus, balanced carefully against the risk of hemorrhagic expansion. He was discharged on triple therapy (dual antiplatelet therapy plus oral anticoagulation) with close outpatient follow-up.

## Outcome and Follow-Up

The patient remained stable without further embolic events or rupture. Repeat imaging at 3 months showed resolution of the thrombus but persistent akinesis of the anterior wall with adverse remodeling, consistent with the poor prognostic implications of large hemorrhagic infarctions.

## Discussion

The advent of primary PCI has established rapid reperfusion as the gold standard for reducing mortality in STEMI.^1^ However, the restoration of blood flow to ischemic tissue is a double-edged sword, capable of inducing reperfusion injury in the form of IMH. Recent data indicate that IMH occurs in up to 40% of STEMI cases after revascularization.^2^ Although IMH is a known driver of adverse remodeling,^3^^,^^4^ this case demonstrates a critical acute mechanical sequela: endocardial disruption.

Classically, LV thrombus is attributed to stasis in an akinetic apex. In this case, significant apical akinesis was present. Although the patient received 14,000 U of intraprocedural heparin followed by 5,000 U every 6 hours post-PCI, the formation of a mobile thrombus anchored specifically to a region of severe IMH raises the possibility of a “double-hit” mechanism: stasis augmented by a structural breach of the endocardium.

Our feature-tracking analysis demonstrates the biomechanics of this failure: a critical mismatch where the remote inferolateral wall contracted vigorously (strain −17.0%) while the infarct core underwent paradoxical thinning (strain +11.5%). This severe “tug of war” generated a shear strain magnitude of 28.5%. To contextualize this magnitude, prior CMR feature-tracking studies in uncomplicated anterior STEMI typically report peak shear strain values in the range of 10% to 15% at the infarct border zone.^5^ The nearly 2-fold increase observed in this case underscores the strong mechanical mismatch in the presence of IMH, which we posit may be sufficient to overcome the structural integrity of the endocardium.

This mechanical hypothesis explains the specific morphology and location of the thrombus, which differed from classic stasis-related clots. Stasis thrombi typically form as broad-based, laminar layers along akinetic walls. In contrast, the thrombus observed here was elliptical and pedunculated, suggesting a specific focal anchor point rather than diffuse layering. Spatially, this anchor coincided with the LV apex. As the thinnest myocardial segment, the apex acts as a structural tipping point where the convergence of severe IMH and maximal mechanical shear strain compromised the endocardial barrier. The hyperdynamic function of the remote myocardium, though compensatory for maintaining cardiac output, paradoxically increases the wall stress at this vulnerable border zone, a biophysical penalty of preserved global function.

The accumulation of extravasated blood creates a hydraulic wedge.^6^ The structural fate of this core is determined by the path of least resistance. Whereas outward pressure can lead to fatal free-wall rupture, the necrotic subendocardium often poses a more fragile barrier. In this inward dissection, the pressure peels back the endocardium, creating a breach in continuity with the ventricular cavity.^7^ This exposes the deep myocardial core, which is rich in tissue factor, triggering immediate local coagulation via the extrinsic pathway.^8^ This thrombogenic nidus is fundamentally different from stasis-derived thrombi; it is anchored to the subendothelial matrix, potentially making it persistent despite anticoagulation. The exposed collagen and tissue factor create a prothrombotic surface that overwhelms the local anticoagulant milieu, necessitating systemic therapy.

## Clinical Implications

This case reveals a pathophysiological parallel to the fibrinolytic era, in which cardiac rupture was often attributed to extensive IMH.^9^ We demonstrate that mechanical reperfusion is not immune to this failure mode. The identification of this mechanism via CMR shifts the paradigm from treating generic post–myocardial infarction stasis to managing a specific structural breach. It presents a therapeutic dilemma: anticoagulation is necessary for the thrombus, but risks expanding the intramural hematoma. In such cases, strict blood pressure control is paramount. Given that hemorrhagic myocardial infarction carries a nearly 3-fold higher risk of in-hospital mortality,^10^ early identification via CMR is crucial. Furthermore, this case underscores the limitations of standard echocardiography, which can visualize the thrombus but cannot resolve the underlying endocardial tear or quantify the IMH. Advanced CMR tissue characterization provides the necessary granular detail to differentiate a simple stasis thrombus from one rooted in structural dissection.^4^ The prognostic weight of this finding is significant; beyond acute mortality, these microvascular injury patterns are strongly associated with adverse long-term clinical outcomes.^11^ Clinicians must therefore view IMH not just as a marker of infarct size, but as an active, dynamic lesion capable of evolving into mechanical complications.

## Conclusions

Severe reperfusion hemorrhage can create a mechanical mismatch and high shear strain that dissects the endocardium. This structural breach acts as a potent thrombogenic nidus, independent of stasis. CMR feature tracking is essential for identifying this high-risk substrate.

## Funding Support and Author Disclosures

Dr Rohan Dharmakumar has received research funding from the NIH (HL133407, HL136578, and HL147133. All other authors have reported that they have no relationships relevant to the contents of this paper to disclose.

## Appendix

Video 1 CMR Long Axis Cine View Showing LV Apical Thrombus Cine imaging reveals a profound mechanical mismatch within the left ventricle. The remote inferior wall exhibits compensatory hyperkinesis, generating significant opposing force against the severely dyskinetic and thinning anterior wall and apex. An irregular, pedunculated thrombus is anchored specifically to this region of maximal systolic stretching.

Video 2 CMR 4-Chamber Cine View Showing LV Apical Thrombus Steady-state free precession (SSFP) cine imaging demonstrates severe dyskinesis of the mid-to-distal interventricular septum and left ventricular apex. High-spatial-resolution CMR clearly delineates a distinct, low-signal-intensity mobile mass tethered directly to the thinned, infarcted apical endocardium.

Video 3 CMR 3-Chamber View Showing LV Apical Thrombus Cine imaging displays the anteroseptal and inferolateral walls, delineating the extensive footprint of the ischemic injury. The irregular, mobile thrombus is visualized protruding into the left ventricular cavity, with its structural nidus precisely localized to the dyskinetic anteroseptal-apical border zone.

Video 4 Transthoracic Echocardiography: Apical 2-Chamber View Cine imaging demonstrates profound anterior and apical akinesis. Near-field artifact and endocardial dropout obscure the anatomical apex, precluding definitive evaluation for an apical echodensity.

Video 5 Transthoracic Echocardiography: Apical 3-Chamber View Cine imaging reveals severe anteroseptal and apical akinesis. An irregular, mobile apical echodensity is visualized, highly suspicious for thrombus, although acoustic limitations obscure its specific endocardial attachment.

Video 6 Transthoracic Echocardiography: Apical 4-Chamber View Cine imaging shows septal and apical akinesis. Apical foreshortening and suboptimal acoustic windows render this view equivocal for the presence of a distinct apical thrombus.

Video 7 Transthoracic Echocardiography: Short-Axis View Mid-ventricular cine confirms severe anterior and anteroseptal dyskinesis, delineating the extent of the left anterior descending artery infarct territory in this section.
